# Using the properties of soil to speed up the start-up process, enhance process stability, and improve the methane content and yield of solid-state anaerobic digestion of alkaline-pretreated poplar processing residues

**DOI:** 10.1186/s13068-014-0160-4

**Published:** 2015-01-12

**Authors:** Yiqing Yao, Yang Luo, Tian Li, Yingxue Yang, Hongmei Sheng, Nolan Virgo, Yun Xiang, Yuan Song, Hua Zhang, Lizhe An

**Affiliations:** Ministry of Education Key Laboratory of Cell Activities and Stress Adaptations, School of Life Sciences, Lanzhou University, Lanzhou, 730000 China; Cuiying Honors College, Lanzhou University, Lanzhou, 730000 China

**Keywords:** SS-AD, PPRs, Alkaline pretreatment, Properties of soil, Process performance, Methane yield, Structural changes

## Abstract

**Background:**

Solid-state anaerobic digestion (SS-AD) was initially adopted for the treatment of municipal solid waste. Recently, SS-AD has been increasingly applied to treat lignocellulosic biomass, such as agricultural and forestry residues. However, studies on the SS-AD process are few. In this study, the process performance and methane yield from SS-AD of alkaline-pretreated poplar processing residues (PPRs) were investigated using the properties of soil, such as buffering capacity and nutritional requirements.

**Results:**

The results showed that the lignocellulosic structures of the poplar sample were effectively changed by NaOH pretreatment, as indicated by scanning electron microscopy and Fourier transform infrared spectra analysis. The start-up was markedly hastened, and the process stability was enhanced. After NaOH pretreatment, the maximum methane yield (96.1 L/kg volatile solids (VS)) was obtained under a poplar processing residues-to-soil sample (P-to-S) ratio of 2.5:1, which was 29.9% and 36.1% higher than that of PPRs (74.0 L/kg VS) and that of experiments without NaOH pretreatment (70.6 L/kg VS), respectively. During steady state, the increase in the methane content of the experiment with a P-to-S ratio of 2.5:1 was 4.4 to 50.9% higher than that of the PPRs. Degradation of total solids and volatile solids ranged from 19.3 to 33.0% and from 34.9 to 45.9%, respectively. The maximum reductions of cellulose and hemicellulose were 52.6% and 42.9%, respectively, which were in accordance with the maximal methane yield. *T*_80_ for the maximum methane yield for the experiments with NaOH pretreatment was 11.1% shorter than that for the PPRs.

**Conclusions:**

Pretreatment with NaOH and addition of soil led to a significant improvement in the process performance and the methane yield of SS-AD of PPRs. The changes in lignocellulosic structures induced by NaOH pretreatment led to an increase in methane yield. For the purpose of practical applications, SS-AD with soil addition is a convenient, economical, and practical technique.

## Background

Solid-state anaerobic digestion (SS-AD) has been used to treat municipal solid waste since the early 1990s, and is the dominant anaerobic digestion (AD) system used in Europe. The total solids (TS) content of SS-AD ranges from 15 to 40% [1,2]. In recent years, various types of lignocellulosic materials and other organic wastes with high solid contents have been treated by SS-AD to produce biogas [3,4]. Compared with liquid-state anaerobic digestion (LS-AD), SS-AD has many advantages. For example, SS-AD requires less energy and water and can treat more organic solids, and the digested residues can be easily handled without dewatering [5,6]. Codigestion of two or more different feedstocks in a single reactor is popular in the field of bioenergy recovery (biogas production), because it offers balanced nutrition for the enhancement of methane yield [7,8]. However, in practice, it is often difficult to collect suitable materials for codigestion with the feedstock of interest, so the operation of SS-AD is difficult to guarantee. As a result, SS-AD of a single feedstock is necessary for practical purposes. The most critical phase of SS-AD of single feedstock is the start-up period. It is well known that the addition of a large amount of inoculum (up to 50%) is beneficial for the acceleration of the start-up of SS-AD [9]. However, for a certain working volume of a reactor, an increase in the proportion of inoculum in mixtures leads to a decrease in the effective working volume [10,11].

To date, a scientific understanding of the SS-AD process is lacking. Despite the large number of existing studies on SS-AD and the rising interest in this technology, very few investigations have been performed on its stability [12,6]. Soil has the ability to maintain a constant pH, which is referred to as its buffering capacity. In systems with a buffering capacity, soil has the ability to neutralize acids and bases [13]. Additionally, soil contains multiple elements required by microorganisms as nutrition, including carbon, nitrogen, phosphorus, potassium, sodium, magnesium, and calcium [13]. Soil is also abundant, conveniently available, and can be handily collected. These features lead to several obvious benefits. First, the problem of process instability of AD can be overcome; process instability is a common phenomenon with SS-AD, especially for the SS-AD of a single feedstock. Second, the SS-AD technique is simplified, because there is no need to codigest with other materials for process stability. Third, the cost will be low because of the abundance and convenient availability of soil. Because of these properties of soil, the application of SS-AD with soil addition is convenient, economical, and practical.

As a type of lignocellulosic biomass, poplar processing residues (PPRs) are good substrates for LS-AD following NaOH treatment [14]. In this study, PPRs were used for methane yield by SS-AD.

The goals of this study were: (1) to examine the influence of soil on the speed of the start-up period, the steady-state conditions, the methane yield, and the methane content of SS-AD with NaOH-pretreated PPRs as substrate; (2) to optimize the conditions of SS-AD; (3) to analyze the benefits of soil utilization on SS-AD.

## Results and discussion

### Pretreatment

#### Degradation of poplar solids after treatment

The results clearly show that the pH value was not stable and varied widely during the 5-day pretreatment period. After that, only a slight variation in pH was observed; the pH remained between 7.1 and 7.5 and was optimum for AD, meaning that there was no need to adjust the pH of mixtures prior to SS-AD. This indicates that 5 days of pretreatment time is optimal; this is shorter than that required for solid-state pretreatment time (21 days) [15]. Therefore, the method of pretreatment in this study is a time-saving and convenient technique.

Table 1 shows the characteristics of the PPRs, inoculum, and soil samples. After 5 days of pretreatment, the contents of cellulose, hemicellulose, and lignin were 49.9%, 24.9%, and 21.6%, respectively. The degradations of cellulose, hemicellulose, and lignin were 9.6%, 4.8%, and 12.7%, respectively. These results indicate that pretreatment with NaOH effectively removed the lignin of the lignocellulose structure. It has been reported that lignin protects cellulose and hemicellulose from degradation and destruction [16].

**Table 1:** **Characteristics of PPRs**, **inoculum**, **and soil**

**Parameter**	**PPRs**	**Inoculum**	**Soil**
TS (%)	83.6 ± 0.0	20.1 ± 0.1	83.3 ± 0.0
VS (%)	89.8 ± 0.3	58.3 ± 0.2	3.8 ± 0.0
TC (%)	45.9 ± 0.1	29.8 ± 0.1	2.6 ± 0.1
TN (%)	0.2 ± 0.0	1.9 ± 0.1	0.06 ± 0.0
H (%)	5.6 ± 0.2	4.0 ± 0.1	0.4 ± 0.0
pH	7.7 ± 0.0	7.3 ± 0.0	6.9 ± 0.0
Cellulose (%)	55.3 ± 0.3	39.0 ± 0.8	ND
Hemicellulose (%)	26.1 ± 0.7	27.1 ± 0.7	ND
Lignin (%)	24.3 ± 1.4	ND	ND

TS: total solids; VS: volatile solids; TC: total carbon; TN: total nitrogen; H: hydrogen.

ND: not determined.

#### Scanning electron microscopy (SEM)

**Figure 1:**
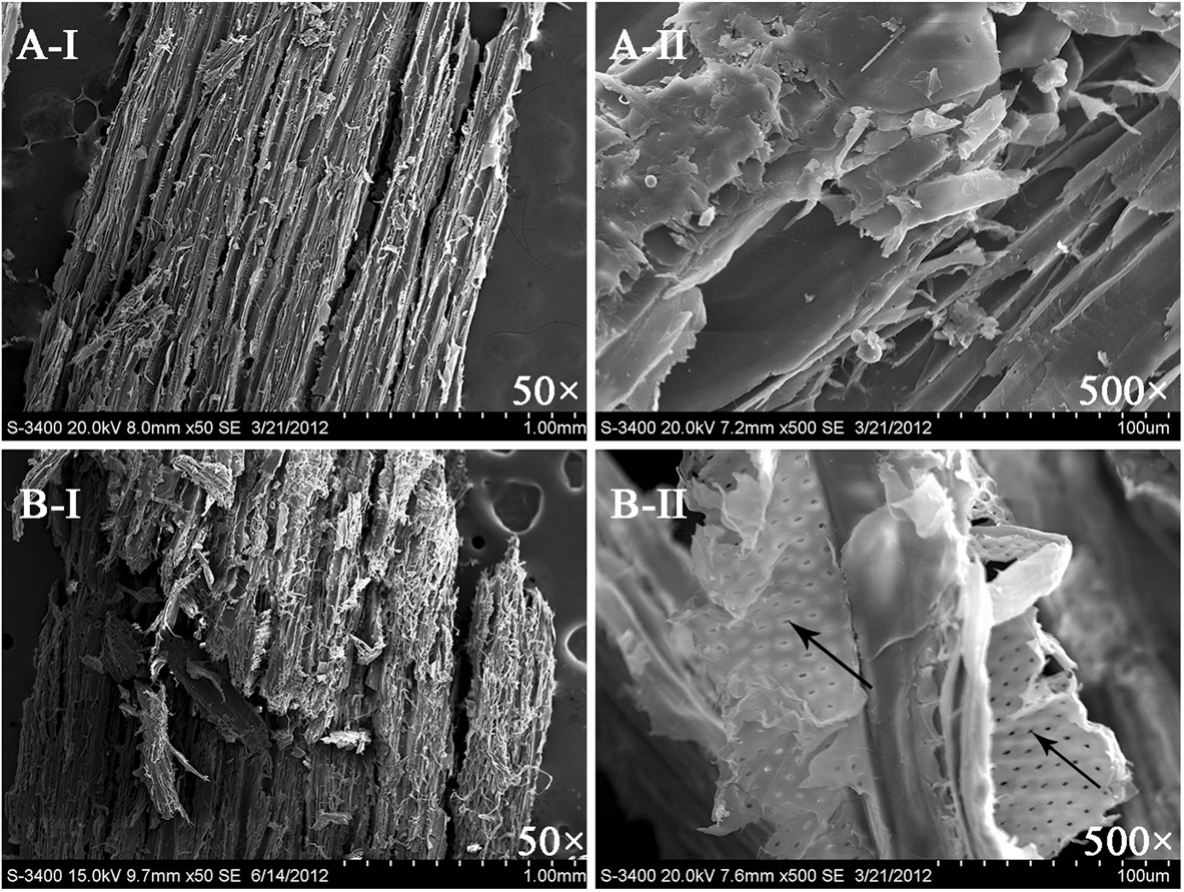
**SEM photos of PPRs.** Untreated PPRs, 50 × **A**-I, 500 × **A**-II; PPRs pretreated with 3.0% NaOH, 50 × **B**-I, 500 × **B**-II.

Figure 1 shows the SEM images of non-NaOH-pretreated and NaOH-pretreated samples at magnifications of 50× and 500×. The compact texture and smooth surface of the raw samples can be seen in Figure 1A-I and 1A-II, respectively. The structure of the fiber of the NaOH-pretreated samples was damaged and many holes formed on the surface, indicating that most of the lignin and hemicellulose was removed. The removal of lignin and hemicellulose was beneficial for the biodegradability of lignocellulosic substrate and for subsequent AD [14].

#### Fourier transform infrared (FTIR) spectra

**Figure 2:**
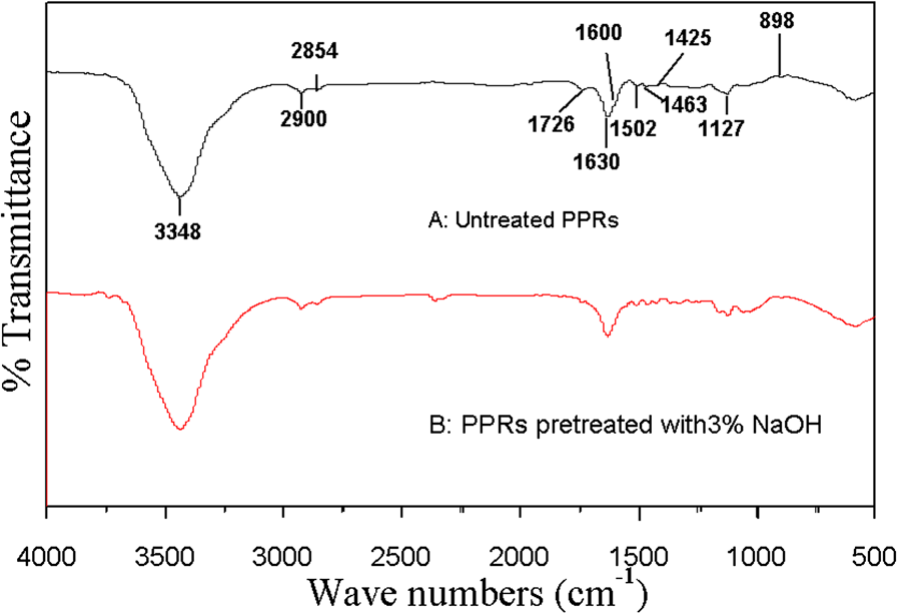
**FTIR spectra of PPRs. A**: Untreated PPRs; **B**: PPRs pretreated with 3.0% NaOH.

FTIR spectroscopy is a useful method for investing the chemical and constituent changes of lignocellulose materials [17]. As shown in Figure 2, after NaOH pretreatment, the intensity of the peak at 1726 cm^-1^ (carbonyl C = O stretching) decreased, indicating that the lignin side chains were cleaved [18,19]. The peaks ranging from 1502 to 1600 cm^-1^ represented aromatic skeletal vibration; the reduction in intensities of these peaks illustrate that lignin was dissolved effectively by 3% NaOH. In the band at 898 cm^-1^, which was assigned to acetyl groups, the intensity of absorption decreased, indicating that the groups were cleaved [20-22]. This proves that the 3% NaOH pretreatment can remove the lignin and effectively disrupt the structure of lignocellulose, and these structural changes indicate that subsequent contact between microorganisms and the substrate during AD will be favorable [14].

### Anaerobic digestion

#### Methane yield

**Figure 3:**
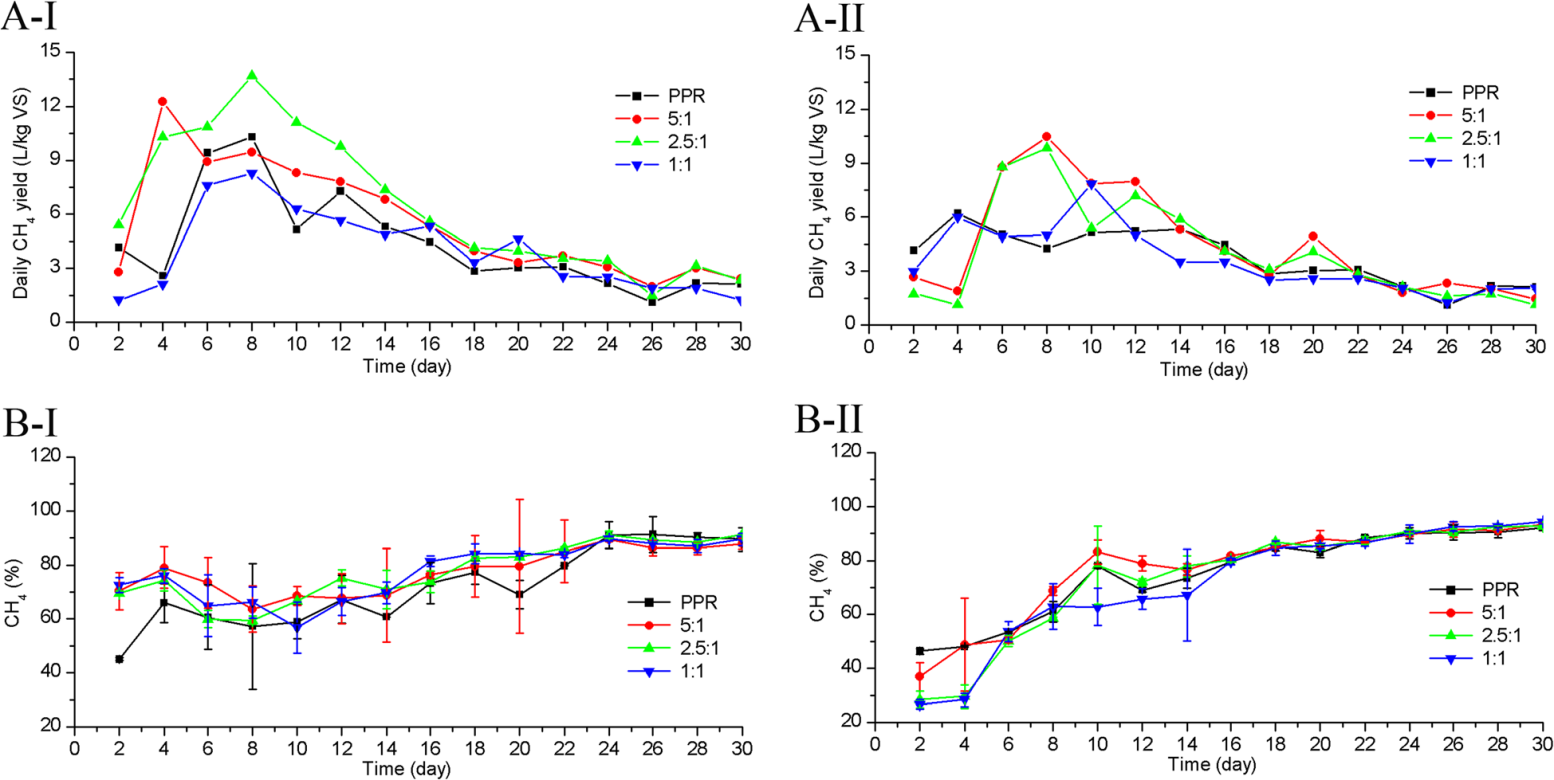
**Methane yield for PPRs and for P-to-S ratios of 5:1, 2.5:1, and 1:1. A**: Daily methane yield (L/kg VS), **B**: Methane content (%); I: NaOH pretreatment, II: non-NaOH pretreatment.

The daily methane yields of the NaOH-pretreated PPRs and the control (non-NaOH-pretreated PPRs) are presented in Figure 3A. The trends of the daily methane yields for the experiments with soil addition were different from those of the PPRs (Figure 3A-І and 3A-Π), especially for experiments with poplar processing residues-to-soil sample (P-to-S) ratios of 5:1 and 2.5:1. As shown in Figure 3A-І, the daily methane yields of experiments with soil addition continuously increased in the initial period of SS-AD, a trend that was different from that observed for the PPRs. The daily methane yields for P-to-S ratios of 5:1 and 2.5:1 were higher than those for the PPRs and the 1:1 ratios. Their peak values (12.3 L/kg volatile solids (VS) and 13.7 L/kg VS) were reached on days 4 and 8, and were 9.8% and 22.3% higher, respectively, than those of the PPRs (11.2 L/kg VS). For the daily methane yield of PPRs, according to Buyukkamaci and Filibeli [23], the accumulation of volatile fatty acids (VFAs) or the high level of VFAs present during the initial period of SS-AD may inhibit methanogenesis or disrupt the biomass community balance to a certain extent, because the level of VFAs indicates the metabolic state of acetogens and acetoclastic methanogens [6,23,24]. The phenomenon of acidification during the initial period of both SS-AD and LS-AD is common. Zhu *et al.* [16] investigated the SS-AD of corn straw after alkaline pretreatment and found that the lag phase appeared in the initial period [16]. When fallen leaves were used for methane yield through simultaneous alkaline treatment, the lag phase at the initial stage was long at a substrate-to-inoculum ratio of 6.2:1 [25]. Xu and Li [26] studied the effect of SS-AD of expired dog food and corn stover on methane yield and observed a long lag phase [26]. Brown and Li [9] observed a decline in methane yield in the initial period of SS-AD of yard waste and food waste [9]. A long lag phase also appeared during LS-AD of corn stover [15]. These results indicate that soil addition can maintain a stable increase in daily methane yield at the set-up stage of SS-AD due to its buffering capacity. In soil, the strength of absorption of different cations is generally regarded to occur in the following order:

When acidity increases (pH decreases), more H^+^ ions are attached to the colloids, while other cations are pushed away from the colloids [27]. Multiple elements are present in soil, and are required for microorganisms as nutrition [13]. However, for experiments with a P-to-S ratio of 1:1, the addition of large amounts of soil might inhibit contact between anaerobic biomass and biodegradable organic matter, leading to low daily methane yield throughout the SS-AD process. Additionally, the initial daily methane yields of experiments with soil addition continuously increased. By contrast, there was a temporary decline after the first 2 days of SS-AD for PPRs; this can be attributed to the dissipation of substrate readily available for microbial decomposition, and a large fluctuation that appeared after the peak value of daily methane yield (day 8), indicating instability of the SS-AD process [28]. Although the daily methane yield of PPRs throughout SS-AD was low, the process evolved satisfactorily. In addition to the specific characteristics of PPRs (such as a high pH of 7.7), the presence of toxic agents like free ammonia that diffuse differently from those of LS-AD and inhibition phenomena are avoided under conditions of high solid content [6]. Otherwise, when the solid content is low, an inhibitory phenomenon resulting from the accumulation of VFAs is common [29]. According to Wang *et al.* [29], the VFA concentrations in high solid anaerobic digestion (HS-AD) or dry AD are much higher than those in LS-AD, which are considered to be inhibitory to the LS-AD process [29]. Similarly, for the control (Figure 3A-II), the daily methane yields for experiments with P-to-S ratios of 5:1 and 2.5:1 were higher than those for the PPRs and experiments with a P-to-S ratio of 1:1 during days 6 to 14. The daily methane yields for P-to-S ratios of 5:1 and 2.5:1 reached peak values of 10.5 L/kg VS and 9.8 L/kg VS, respectively, on day 8. Both of the peak values were lower than those of the corresponding experiments with NaOH pretreatment. For the experiments without NaOH pretreatment, temporary declines were observed during the initial stage of SS-AD; this occurred because of the small amount of available organics for microbial decomposition and the recalcitrance created by the complex structure of native lignocellulosic biomass to enzymes [30]. Fluctuations in the methane yields also appeared in the control experiment. For AD, a fluctuation of methane yield is common due to the acidification phenomenon during the initial period AD. Liew *et al.* [25] investigated the process performance of SS-AD of fallen leaves through simultaneous NaOH treatment, and observed that the fluctuations in methane yield were large for control and NaOH-treated experiments [25]. Cioabla *et al.* [31] compared the biogas yields of a mix batch and a wheat bran substrate, and observed large fluctuations during the AD process for both batches [31]. In a study by Aymerich *et al.* [6], agro-industrial waste and sewage sludge were used for methane yield under high-solid conditions; methane yield processes were very unstable in all the experiments [6]. After NaOH pretreatment, the methane yield from corn straw at solid state was not steady in the work [16]. These results prove that soil addition can enhance the stability of the SS-AD process of NaOH-pretreated PPRs.

For experiments with NaOH pretreatment (Figure 3B-І), the methane content for the experiments with soil addition were higher than those for those without soil addition and were superior to 50% on day 2 of SS-AD, indicating that the SS-AD process was in a stable state [9,16]. The methane content ranged from 60.0 to 91.1%. The methane content of PPRs was more than 50% higher on day 4 of SS-AD and more than 70% higher until day 18 of SS-AD, and there were clear fluctuations of methane yield over the course of the SS-AD. This was in accordance with the corresponding daily methane yield (Figure 3A-І). For AD, the methane content is usually less than 75%, and the time required to reach steady state is usually long. For example, Zhu *et al.* [16] sudied the methane yield of NaOH-pretreated corn straw at solid state. The methane content reached steady state on day 9, which was 7 days longer than the time required in experiments with NaOH pretreatment and soil addition, and the methane content was less than 70%, which was also lower than those for experiments with NaOH pretreatment and soil addition [16]. Xu *et al.* [32] compared different liquid AD effluents as inocula and nitrogen sources for solid-state batch AD of corn straw, and observed that steady state was reached on day 5 [32]. Aymerich *et al.* [6] analyzed the stability of HS-AD of agro-industrial waste and sewage sludge, and in all but one experiment, steady state was reached between day 5 and day 15 [6]. A long time was also required to reach a methane content of 50% in the studies of Brown and Li [9] (6 to 16 days) and Xu and Li [26] (5 to 10 days), and the methane contents were all less than 75% [9,26]. These results underline the idea that soil addition following NaOH pretreatment reduced the amount of time required to reach steady state and increased the methane content. The increase in the methane content for the experiment with a P-to-S ratio of 2.5:1 was 4.4 to 50.9% higher than that of the PPRs during the steady-state period of SS-AD. Considering the relatively low standard deviation, the repeatability and reproducibility of biogas quality under a P-to-S ratio of 2.5:1 were high. When non-NaOH-pretreated PPRs were used as a substrate for SS-AD (Figure 3B-II), the methane content in the experiments with soil addition reached steady state (≥50%) on day 6; this represents a delay of 4 days compared with the experiments with NaOH pretreatment (Figure 3B-І). The methane content during the steady-state period ranged from 50.2 to 90.5%. This indicates that NaOH pretreatment helped improve the methane content and accelerate the achievement of steady state; a similar phenomenon was observed by Shao *et al.* [33]. The methane content was lowest under a P-to-S ratio of 1:1 during the first 14 days of SS-AD. On one hand, the raw materials without pretreatment could not be degraded efficiently by fermentative bacteria, which led to the low methanogenesis efficiency [16,30]. On the other hand, the addition of large amounts of soil inhibited the utilization of biodegradable organic matter by methanogens because of the low efficiency of contact between substrate and methanogens, leading to a low methane content.

In general, NaOH pretreatment effectively improved the total methane yields. Similar results were reported by Zheng *et al.* [15], who observed a significant increase in methane yield with NaOH addition at a high loading of corn straw [15]. Zhu *et al.* [16] studied SS-AD of corn straw with NaOH pretreatment; their results demonstrated that the maximum biogas yield was 37.0% higher than that of untreated corn straw [16]. For the NaOH-pretreated experiments, the total methane yields for the PPRs and for the experiments with P-to-S ratios of 5:1, 2.5:1, and 1:1 were 74.0 L/kg VS, 79.2 L/kg VS, 96.1 L/kg VS, and 56.3 L/kg VS, respectively. The maximum methane yield was obtained under a P-to-S ratio of 2.5:1, which was 29.9%, 21.2%, and 71.4% higher than those of the PPRs and the experiments with ratios of 5:1 and 1:1, respectively; the enhancements in methane yield were significant (*P* <0.05). Additionally, the lowest methane yield was obtained for the experiment with a P-to-S ratio of 1:1, because of the inhibition of contact between feedstock and anaerobic biomass. Therefore, soil addition in the appropriate proportion significantly improved the total methane yield of NaOH-pretreated PPRs. The maximum methane yield (96.1 L/kg VS) obtained in this study was 17.1% higher than that obtained from NaOH-pretreated fallen leaves (82.0 L/kg VS) [25]. For the control, the total methane yields for the PPRs and the experiments with P-to-S ratios of 5:1, 2.5:1, and 1:1 were 54.3 L/kg VS, 70.6 L/kg VS, 60.5 L/kg VS, and 53.2 L/kg VS, respectively; these values were 26.6%, 10.9%, 37.0%, and 5.5% lower than those of the corresponding experiments with NaOH pretreatment. This indicates that soil addition in the appropriate proportion also significantly improved the total methane yield of non-NaOH-pretreated PPRs. It also shows that NaOH pretreatment increased the methane yield. The difference between the total methane yields for the PPRs and the experiments with a P-to-S ratio of 1:1 was not significant (*P* >0.05). However, the maximum methane yield (70.6 L∕kg VS) was achieved under a P-to-S ratio of 5:1, which was 30.0% and 16.7% higher than those of the PPRs and the experiment with a P-to-S ratio of 2.5:1, respectively; these differences were significant (*P* <0.05). This indicates that NaOH pretreatment significantly increased the total methane yield of PPRs, and soil addition in the appropriate proportions effectively improved the total methane yield of both NaOH-pretreated and non-NaOH-pretreated PPRs.

After SS-AD, the discharges of digested residues from experiments with soil addition were more easily obtained than those without soil addition, reflecting the lubricating property of the soil.

#### Degradations of TS, VS, cellulose, and hemicellulose

The results demonstrate that the maximum reductions in TS were obtained in PPRs with NaOH and without NaOH pretreatments (Table 2). The reason for this is the low VS content of soil (3.8%). As a result, when the P-to-S ratio was reduced from 5:1 to 1:1, or the proportion of the amount of soil addition in the mixtures was increased, reductions of TS decreased. The minimum reduction of TS (3.1%) was obtained with a P-to-S ratio of 1:1 in the control. The TS reductions for the experiments with NaOH pretreatment were higher than those of the controls. For both the NaOH-pretreated and non-NaOH-pretreated experiments, the VS reductions for the experiments with soil addition were generally in line with the total methane yields. As the VS reduction, cellulose, and hemicellulose reductions were in line with the total methane yields (Table 3), higher cellulose and hemicellulose reductions were associated with higher total methane yields. For the experiments with NaOH pretreatment, the greatest reductions of cellulose and hemicellulose were 52.6% and 42.9%, respectively, which were 28.3% and 62.5% higher than that of the PPRs; for the experiments without NaOH pretreatment, the greatest reductions of cellulose and hemicellulose were 45.8%, and 42.5%, respectively, which were 3.4% and 64.7% higher than that of the PPRs.

**Table 2:** **Total solids and volatile solids degradation** (%) **in the mixtures following solid**-**state anaerobic digestion**

**Parameter**		**Control**	**5** **:** **1**	**2.5** **:** **1**	**1** **:** **1**
TS	Pretreatment	34.0 ± 3.6	21.5 ± 2.1	19.3 ± 0.0	20.9 ± 1.3
	No pretreatment	33.0 ± 2.2	15.0 ± 3.1	5.7 ± 2.3	3.1 ± 0.9
VS	Pretreatment	37.5 ± 3.7	43.1 ± 1.7	45.9 ± 1.8	36.6 ± 0.0
	No pretreatment	34.9 ± 4.7	42.5 ± 4.3	40.3 ± 3.9	31.3 ± 3.5

*Note*: Degradation (%) of TS and VS were calculated after determination of the TS and VS contents of mixtures before and after anaerobic codigestion.

TS: total solids; VS: volatile solids.

**Table 3:** **Degradation** (%) **of cellulose and hemicellulose of the mixtures following solid**-**state anaerobic digestion**

**Parameter**		**Control**	**5** **:** **1**	**2.5** **:** **1**	**1** **:** **1**
TS	Pretreatment	41.0 ± 7.1	50.1 ± 7.5	52.6 ± 1.7	13.6 ± 2.2
	No pretreatment	44.3 ± 2.2	45.8 ± 6.5	32.1 ± 3.8	44.7 ± 1.5
VS	Pretreatment	21.3 ± 1.0	26.4 ± 1.6	42.9 ± 7.1	7.8 ± 0.2
	No pretreatment	25.8 ± 3.9	42.5 ± 1.3	38.7 ± 2.1	13.8 ± 3.1

*Note*: Degradation (%) of cellulose and hemicellulose were calculated after determination of the TS and VS contents of mixtures before and after anaerobic codigestion.

TS: total solids; VS: volatile solids.

Based on the above results, more methane was produced when the PPR consumption was higher.

#### Technical digestion time

The technical digestion time (*T*_80_) is used as an indicator of substrate biodegradability [15,34]. In this study, the SS-AD lasted 30 days, and *T*_80_ was calculated after SS-AD (Table 4). It was observed that *T*_80_ for the experiments with NaOH pretreatment was 10.0 to 22.2% shorter than that for the experiments without NaOH pretreatment. These results further indicate that PPRs became more accessible and more readily biodegradable following NaOH pretreatment. This is in agreement with Zheng *et al.* [15]. For the experiments with NaOH pretreatment, *T*_80_ for the experiment with a P-to-S ratio of 2.5:1 was 11.1% shorter than those of the others. For the control, the *T*_80_ values of the experiments with P-to-S ratios of 5:1 and 2.5:1 were 10.0% shorter than those of the other experiments. These results further indicate that soil addition in appropriate proportions could improve the biodegradability and accessibility of PPRs. The result demonstrates that NaOH pretreatment and soil addition could bring economic benefits by shortening the digestion time and thus increasing the treatment capacity.

**Table 4:** **The technical digestion time for experiments with and without NaOH pretreatment**

	**Control**	**5** **:** **1**	**2.5** **:** **1**	**1** **:** **1**
Pretreatment	18 ± 3	18 ± 2	16 ± 2	18 ± 3
No pretreatment	20 ± 2	18 ± 1	18 ± 4	20 ± 1

### Feasibility in large-scale applications

For practical applications, the cost of AD must be taken into account. Soil can be handily collected, and is abundant and conveniently available. In general, the dose of NaOH required for pretreatment before AD is not low. Results from Liew *et al.* [25] demonstrated that the optimal NaOH load was 3.5%, while higher NaOH loading (5.0%) was reported by Zhu *et al.* [25,16]. In this study, the dose of NaOH was only 3.0%, and the pretreatment was conducted at ambient temperature without extra energy input. Thus, the cost of NaOH used for pretreatment in our study is lower than those of previous studies. Steadman [35] stated that the simplest type of anaerobic digester is a batch digester [35]. The Oregon Department of Energy compared three types of digesters (a covered-lagoon digester, a complete-mix digester, and a plug-flow digester) and found that the batch digester was the least expensive [36]. The SS-AD was operated under mesophilic rather than thermophilic temperatures; additionally, low-cost heat produced as waste heat by gas engines could be used as the energy source for maintaining the operating temperature of SS-AD, and this is being done at some full-scale biogas plants [37].

Based on the above information, the fermentation technique examined in this study is feasible for large-scale applications.

## Conclusions

The effectiveness of soil addition on the process performance and the methane yield of SS-AD of PPRs after NaOH pretreatment is obvious: the start-up stage was hastened, the steady state was enhanced, methane content and methane yield were improved, the efficiency of the working volume utilization was improved because of the limited amount of soil added in the SS-AD mixtures, and the treatment capacity was improved because of the shortened *T*_80_. Based on these significant advantages, it is necessary to apply the strategy used in this study to other lignocellulosic biomasses, especially for agricultural biomass, such as wheat straw, corn stalk, and rice straw, because of their large amounts. In terms of effectiveness, economy, and convenience, if the concept can be realized, it will undoubtedly greatly help the application and popularization of SS-AD in practice.

## Methods

### Feedstock and inoculum

The PPRs were obtained from a wood processing factory located in a suburb of the city of Jiuquan, Gansu, China. The samples were ground into 6- to 12-mm particles by a hammer mill (RT-34, Beijing WeiBo Chuang, China). The resultant PPRs were stored at -20°C prior to use. The inoculum was obtained from a biogas plant digesting manure in the city of Dingxi, Gansu, China. Soil samples were collected from the campus of Lanzhou University and were ground into powder.

### Pretreatment

In this study, a dose of 3.0% NaOH (based on dry matter) was used, which was based on the results of wet state AD [14]. The moisture content was 80%. Experiments without NaOH addition were used as the control. All the prepared samples were kept at ambient temperature (20 ± 1°C). Samples used for chemical composition analysis were collected after pretreatment and dried at 60°C for 48 h in an electric oven. The dried samples were kept in a refrigerator [38].

### Anaerobic digestion

Batch mode on a laboratory scale was adopted in this study. The volume of each digester was 2 L. Forty grams of untreated and 40 g of 3.0% NaOH-pretreated samples were put into each digester (based on VS). Then, 83 g of inoculum per 1 L of digester was added. NH_4_Cl was dissolved in deionized water as a nitrogen source, which was placed into each digester to obtain a C-toN ratio of 25:1; the amount of NH_4_Cl added to each digester was 2.2 g/L [31]. For the experiments with NaOH pretreatment and without NaOH pretreatment, the last step was to add powdered soil into the digesters to obtain P-to-S ratios of 5:1, 2.5:1, and 1:1. The amount of soil addition was 8.9 g, 17.8 g, and 44.5 g, respectively; the amounts were calculated based on dry weight, and no soil was added to the SS-AD of the PPRs alone. Nitrogen gas was used to flush the headspace of the digesters for about 5 min per digester to obtain anaerobic conditions; then the digesters were capped tightly with rubber stoppers. The prepared digesters were incubated at 37°C (mesophilic temperature) without shaking, which was the optimal temperature for AD [31]. Each condition was repeated in triplicate. For the PPRs, and experiments with P-to-S ratios of 5:1, 2.5:1, and 1:1, the TS values were 61.2 g, 69.2 g, 77.2 g, and 101.2 g, respectively, and the VS values were 49.6 g, 49.9 g, 50.2 g, and 51.1 g, respectively.

### Analytical methods

#### Chemical composition analyses

TS and VS were measured according to the procedures of the APHA standard [39]. An elemental analyzer (vario EL cube, Elementar Analysensysteme GmbH, Germany) was used to determine total carbon, total nitrogen, and total hydrogen. Prior to the pH determination by pH meter (PB-21, Sartorius, Goettingen, Germany), the poplar samples were prepared by suspending 5 g of wet sample into 50 ml of distilled water [39]. The pH of the soil sample was measured on a 1:5 ratio of sample to water after shaking for 30 min [40]. According to the methods of Van Soest *et al.* [41], the cellulose, hemicellulose, and lignin contents were determined [41]. The data obtained, except for the pH, were based on dry weight.

#### Biogas analyses

Water displacement was used to record the biogas yield every 2 days. After SS-AD, the total biogas volume was calculated. The biogas composition was analyzed with a gas chromatograph (Agilent Technologies, 7890A, Wilmington, DE, USA) equipped with a thermal conductivity detector and a 25 m × 530 μm × 20 μm chromatographic column. Hydrogen was used as the carrier gas, and the flow rate was 35 ml/min. The temperatures of the injector port and detector were 75°C and 150°C, respectively. The composition of the standard gas (YQD-09, Qingdao HuaQing Co., Shandong, China) was 30.1% N_2_, 39.9% methane, and 30.0% CO_2_.

#### SEM

After the samples were sputter-coated with a thin layer of gold, the microscope photos of untreated and pretreated biomass were taken with a Model S-3400 N SEM (Hitachi, Japan).

#### FTIR spectra

The chemical structures of the samples were analyzed by an FTIR system (Nexus 670, Nicolet, USA) with a resolution of 4 cm^-1^. Conditions of 32 scans from 4000 to 500 cm^-1^ were used to obtain the spectra.

#### Statistical analysis

The software SPSS 19.0 was used for the analysis of standard deviations.

## Abbreviations

AD: anaerobic digestion
FTIR: Fourier transform infrared
HS-AD: high-solid anaerobic digestion
LS-AD: liquid-state anaerobic digestion
PPRs: poplar processing residues
P to S: poplar processing residues to soil sample
SEM: scanning electron microscopy
SS-AD: solid-state anaerobic digestion
TS: total solids
VFAs: volatile fatty acids
VS: volatile solids
